# Performance of Diagnostic Algorithms in Patients With Invasive Pulmonary Aspergillosis

**DOI:** 10.1093/cid/ciae633

**Published:** 2024-12-20

**Authors:** Stefan Hatzl, Christina Geiger, Lisa Kriegl, Laura Scholz, Alexander C Reisinger, Philipp Kreuzer, Sonja Fruhwald, Albert Wölfler, Andreas Reinisch, Dirk von Lewinski, Gernot Schilcher, Martin Hoenigl, Philipp Eller, Robert Krause

**Affiliations:** Intensive Care Unit, Department of Internal Medicine, Medical University of Graz, Graz, Austria; BioTechMed-Graz, Graz, Austria; Division of Infectious Diseases, Department of Internal Medicine, Medical University of Graz, Graz, Austria; BioTechMed-Graz, Graz, Austria; Division of Infectious Diseases, Department of Internal Medicine, Medical University of Graz, Graz, Austria; Emergency Department, Department of Internal Medicine, Medical University of Graz, Graz, Austria; Intensive Care Unit, Department of Internal Medicine, Medical University of Graz, Graz, Austria; Emergency Department, Department of Internal Medicine, Medical University of Graz, Graz, Austria; Division of Anesthesiology and Intensive Care Medicine II, Department of Anesthesiology and Intensive Care Medicine, Medical University of Graz, Graz, Austria; Division of Hematology, Department of Internal Medicine, Medical University of Graz, Graz, Austria; Division of Hematology, Department of Internal Medicine, Medical University of Graz, Graz, Austria; Department of Blood Group Serology and Transfusion Medicine, Medical University of Graz, Graz, Austria; Division of Cardiology, Department of Internal Medicine, Medical University of Graz, Graz, Austria; Department of Internal Medicine, Landeskrankenhaus Südsteiermark, Wagna, Austria; BioTechMed-Graz, Graz, Austria; Division of Infectious Diseases, Department of Internal Medicine, Medical University of Graz, Graz, Austria; Intensive Care Unit, Department of Internal Medicine, Medical University of Graz, Graz, Austria; BioTechMed-Graz, Graz, Austria; Division of Infectious Diseases, Department of Internal Medicine, Medical University of Graz, Graz, Austria

**Keywords:** invasive pulmonary aspergillosis, ICU, host factors, classification, IPA

## Abstract

**Background:**

Invasive pulmonary aspergillosis (IPA), once limited to immunocompromised patients, is now a severe complication in critically ill ICU patients without classic risk factors. Due to the difficulty of obtaining histological evidence, diagnosis relies on poorly tested algorithms in real-world settings.

**Methods:**

We conducted a retrospective multicenter (n = 9) cohort study including 202 patients with IPA. Patients were classified using a multistep process based on the EuropeanOrganization- for-the-Research-and-Treatment-of-Cancer/Mycosis-Study Group (EORTC-MSG), Invasive-Fungal-Diseases-in-Adult-Patients-in-Intensive-Care-Unit (FUNDICU), Aspergillus-ICU (Asp-ICU), and Asp-ICU with biomarkers (Asp-ICU-BM) criteria. We then evaluated the predictive performance of these criteria against the clinical cohort and histologically proven cases.

**Results:**

Among 202 patients, 78 had EORTC-MSG host factors and were classified accordingly, with EORTC-MSG criteria achieving 100% agreement in identifying clinical and histologically proven cases. In 112 ICU patients without EORTC-MSG host factors, overall agreement was 53% for FUNDICU, 4% for Asp-ICU, and 26% for Asp-ICU-BM versus the clinical cohort. Validation against histologically proven cases showed FUNDICU had 44% sensitivity and 75% specificity, Asp-ICU 6% sensitivity and 100% specificity, and Asp-ICU-BM 28% sensitivity and 63% specificity. Adding acute respiratory distress syndrome (ARDS) and post-cardiac surgery to the FUNDICU criteria improved sensitivity to 97% with a specificity of 63%. The remaining 12 patients lacked EORTC-MSG host factors and were not in the ICU, highlighting a novel classification system.

**Conclusions:**

EORTC-MSG and FUNDICU IPA classification systems are useful for the assignment of most patients with IPA. Incorporating postoperative complications after cardiac surgery and ARDS enhanced the diagnostic accuracy of FUNDICU.

## Teaser

Invasive pulmonary aspergillosis is increasingly detected in ICU patients without traditional risk factors, making diagnosis difficult. While EORTC-MSG criteria worked well for immunocompromised patients, adding acute respiratory distress syndrome and post-cardiac surgery to the FUNDICU criteria improved diagnostic accuracy.

## Graphical Abstract

**Graphical Abstract:**
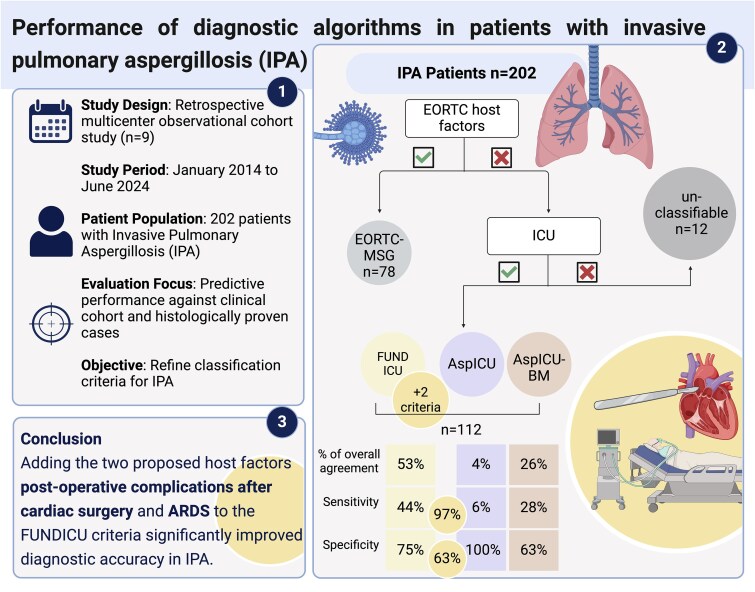

Aspergillus is a common mold encountered in the environment, primarily affecting the lungs through the airways. While most people can eliminate it with a healthy immune system, severely immunocompromised individuals are at risk of invasive pulmonary aspergillosis (IPA) [1–3]. Emerging evidence suggests that IPA is not limited to traditional immunocompromised patients but can also affect those with influenza or coronavirus disease 2019 (COVID-19), particularly in the intensive care unit (ICU) setting [4, 5]. Since most cases of influenza-associated pulmonary aspergillosis (IAPA) and COVID-19–associated aspergillosis (CAPA) occur in critically ill patients, the ICU has become a key area for fungal diagnosis and treatment [6, 7]. A study found an IPA incidence of 6.9% among ICU patients, 70% of whom lacked traditional risk factors like hematologic diseases [8]. In addition to viral pneumonia, other ICU-related risk factors include prolonged corticosteroid use (≥0.3 mg/kg corticosteroids for ≥3 weeks), liver failure, respiratory diseases, cardiovascular disease, diabetes, and sepsis-associated immuno-paralysis [8–10]. The European Organization for the Research and Treatment of Cancer/Mycosis Study Group (EORTC-MSG) criteria, established in 2002 and recently updated, remain restrictive for ICU patients and those without traditional risk factors [11]. To address this limitation, the Aspergillosis in Intensive Care Units (Asp-ICU) and biomarker enhanced (Asp-ICU-BM) criteria were developed [12, 13]. The Invasive Fungal Diseases in Adult Patients in Intensive Care Unit (FUNDICU) initiative also provided standardized definitions for diagnosing IPA in critically ill ICU patients [14]. However, comparative investigations and external validation of these algorithms are still lacking, making their effectiveness unclear.

To address this unmet need, we utilized a multicenter cohort of patients clinically diagnosed with IPA to independently validate available diagnostic tools in a real-world setting. Specifically, we evaluated the diagnostic performance and challenges of the FUNDICU, Asp-ICU, Asp-ICU-BM, and EORTC-MSG criteria in this cohort.

## METHODS

### Study Cohort

We conducted a multicenter observational study across 9 clinical centers, enrolling all consecutive adult patients diagnosed with IPA by an infectious disease (ID) consultant in routine clinical care (Supplementary Figure 1). For inclusion in the final analysis, the diagnosis of IPA had to be confirmed by an independent ID and ICU specialist. Patient data were reviewed in a blinded format, and the diagnosis of IPA was either confirmed or rejected. In cases of disagreement, a discussion was held to reach consensus. No patients had to be excluded retrospectively. The study period was 1 January 2014 to 1 June 2024. Uniform patient data collection was conducted as previously described [15]. Laboratory, clinical, and radiological data were extracted from our in-house electronic healthcare database and entered a predefined electronic case report form using REDCap (Research Electronic Data Capture) [16, 17]. The study was approved by the local review board (EK: 32-302ex19/20) and conducted in accordance with the Declaration of Helsinki principles.

### Classification of the IPA Cases

We applied a multistep process to classify IPA cases. First, patients with classical host factors (eg, neutropenia, hematologic malignancy, allogeneic stem cell transplant, solid-organ transplant, prolonged corticosteroid use, recognized T-cell or B-cell immunosuppressants, severe immunodeficiency, or grade III/IV graft-vs-host disease) were classified using EORTC-MSG criteria. These patients, whether treated in or outside an ICU, did not proceed to the next step. Second, patients diagnosed with IPA within an ICU were classified using FUNDICU, Asp-ICU, and Asp-ICU-BM criteria [11–14]. Finally, the remaining patients (non-EORTC, non-ICU) were categorized as unclassifiable IPA (Supplementary Figure 2). Breakthrough fungal infections were classified according to recent European Confederation of Medical Mycology (ECMM)/MSG definitions of breakthrough infections [18].

### Statistical Analysis

All statistical analyses were performed using Stata (Windows version 16.1; StataCorp) and R 4.0.5 (R Foundation for Statistical Computing) according to a prespecified analysis plan. Baseline variables between the traditional host-factor group (EORTC-MSG) and the ICU group (FUNDICU, Asp-ICU, Asp-ICU-BM) were compared using rank-sum, chi-square, and Fisher's exact tests as appropriate.

The primary outcome was the measure of agreement (percent positive agreement [PPA], percent negative agreement [PNA], and percent overall agreement [POA]) between EORTC-MSG, FUNDICU, Asp-ICU, and Asp-ICU-BM using a 2 × 2 contingency table. Patients identified as IPA-positive by FUNDICU, Asp-ICU, and Asp-ICU-BM were collectively grouped as the ICU classification. The validity of each diagnostic algorithm was assessed against pathology results from lung biopsies or necropsies, with predictive values evaluated by sensitivity, specificity, positive predictive value (PPV), and negative predictive value (NPV).

As a secondary outcome, we aimed to enhance diagnostic accuracy by developing a novel algorithm based on the primary outcome data. Algorithm performance was compared using the area under the receiver operating characteristic curve (AUROC) and bootstrapped *P* values with the Stata “rocreg” routine. Survival outcomes, including all-cause mortality and IPA-attributable mortality, were included to describe the cohort. Invasive pulmonary aspergillosis–attributable mortality was defined as death following septic or respiratory deterioration while the IPA was either unresolved or progressing, as indicated by radiological findings or fungal biomarkers [19]. The full dataset and main analysis code are available upon request from the first author.

## RESULTS

### Study Cohort

A total of 202 adults diagnosed with IPA by an ID specialist in routine clinical care were enrolled in our study. In a next step, diagnosis of IPA was confirmed in a blinded review process by an independent ID and ICU specialist. After classification (Supplementary Figure 2), 78 patients were categorized according to the revised EORTC-MSG criteria (irrespective of potential ICU admission), while 112 were classified within ICU-focused classification systems: FUNDICU, Asp-ICU, and Asp-BM. Twelve patients remained unclassifiable due to a lack of EORTC host factors or ICU admission. The median age of the cohort was 63 years (interquartile range [IQR]: 54–71 years); 63 patients (31%) were female. Notably, 47% of patients assigned to the EORTC-MSG group were female, compared to only 21% in the ICU group (*P* < .001). The median body mass index (BMI) was 25.4 kg/m² (IQR: 22.6–28.4 kg/m²).

Most patients (77%, 155/202) were treated in an ICU, including 43 (55%) of the EORTC-MSG group. Patients admitted to the ICU had a median APACHE II (Acute Physiology And Chronic Health Evaluation 2) score of 24 (IQR: 17–31) and a SOFA (Sequential Organ Failure Assessment) score of 7 (IQR: 5–10), indicating severe illness. The EORTC-MSG patients in the ICU had less severe respiratory failure (partial pressure of oxygen in arterial blood/fraction of inspired oxygen [PaO_2_/FiO_2_] of 125 mmHg [IQR: 72–197 mmHg]) compared with the ICU classification group (PaO_2_/FiO_2_ of 101 mmHg [IQR: 71–130 mmHg]) (*P* = .03). All ICU-classified patients exhibited acute respiratory distress syndrome (ARDS), with 50% (55/112) experiencing severe ARDS [20]. Acute respiratory distress syndrome was also prevalent in the EORTC-MSG group, with 55% (43/78) of these patients admitted to the ICU showing respiratory failure. Due to the high rates of severe respiratory failure, most ICU-admitted patients required invasive mechanical ventilation and/or extracorporeal membrane oxygenation (Table 1). Baseline laboratory characteristics are summarized in Table 1. Over a median follow-up of 3.0 years (IQR: 1.1–6.1 years), there were 144 (71%) deaths in the cohort, with 111 (55%) attributed to IPA (Supplementary Figure 3). The distribution of IPA cases varied significantly during the study (Supplementary Figures 4 and 5).

**Table 1.** Baseline Characteristics of the Cohort

Variable	Total Cohort (N = 202)	ICU (n = 112)	EORTC (n = 78)	No Class (n = 12)
Age, y	63 [54–71]	63 [53–70]	61 [53–69]	70 [63–77]
Female gender, n (%)	63 (31%)	24 (21%)	37 (47%)	2 (17%)
BMI, kg/m²	25.4 [22.6–28.4]	26.3 [23.7–29.4]	24.6 [21.6–26.3]	23.5 [20.4–24.9]
ICU characteristics				
ICU admission, n (%)	155 (77%)	112 (100%)	43 (55%)	0 (0%)
APACHE II score	24 [17–31]	25 [17–33]	27 [21–34]	N/A
SOFA	7 [5–10]	7 [4–10]	7 [5–10]	N/A
PaO_2_/FiO_2_	102 [71–148]	101 [71–130]	125 [72–197]	N/A
ARDS				
No ARDS	47 (23%)	0 (0%)	35 (45%)	12 (100%)
Mild	19 (9%)	10 (9%)	9 (11%)	0 (0%)
Moderate	64 (32%)	47 (42%)	17 (22%)	0 (0%)
Severe	72 (36%)	55 (49%)	17 (22%)	0 (0%)
Ventilatory support				
HFNC	3 (2%)	0 (0%)	3 (7%)	0 (0%)
NIV	14 (9%)	9 (8%)	5 (12%)	0 (0%)
IV	114 (74%)	81 (72%)	33 (77%)	0 (0%)
vv-ECMO	24 (15%)	22 (20%)	2 (4%)	0 (0%)
Laboratory findings				
Leukocytes, G/L (10^9^/L)	9.4 [4.6–14.6]	11.8 [7.8–16.3]	4.5 [1.1–9.0]	10.3 [16.8–7.3]
Neutrophils, G/L	8.0 [3.2–12.0]	10.5 [6.8–13.7]	3.0 [0.5–8.4]	6.7 [5.3–15.3]
Lymphocytes, G/L	0.6 [0.3–1.0]	0.7 [0.4–1.3]	0.4 [0.1–0.8]	0.8 [0.6–1.6]
Hemoglobin, g/dL	9.8 [8.9–11.8]	10.0 [8.9–12.4]	9.5 [8.5–10.8]	12.6 [9.9–13.8]
Platelets, G/L	148 [67–235]	177 [90–260]	86 [28–188]	200 [139–276]
CRP, mg/L	116 [53–201]	135 [73–201]	92 [30–214]	66 [40–89]
PCT, ng/mL	0.91 [0.3–4.0]	1.4 [0.4–5.8]	0.7 [0.3–2.8]	0.1 [0.1–0.6]
Bilirubin, mg/dL	0.7 [0.4–1.76]	0.7 [0.5–1.79]	0.7 [0.4–2.0]	0.7 [0.5–0.9]
Creatinine, mg/dL	1.3 [0.8–7.0]	1.8 [0.9–7.0]	1.1 [0.8–3.8]	0.9 [0.7–1.1]
Outcomes				
Response of IPA, n (%)				
No	109 (53%)	66 (59%)	38 (49%)	5 (41%)
Stable disease	8 (4%)	3 (3%)	4 (5%)	1 (8%)
Yes	85 (42%)	43 (38%)	36 (46%)	6 (50%)
Deceased during IPA, n (%)	111 (55%)	66 (59%)	40 (51%)	5 (42%)
Deceased at data cutoff, n (%)	144 (71%)	77 (69%)	58 (74%)	9 (75%)

Values are medians (interquartile range) unless otherwise indicated.

Abbreviations: APACHE II, Acute Physiology And Chronic Health Evaluation 2; ARDS, acute respiratory distress syndrome; BMI, body mass index; CRP, c-reactive protein; EORTC, European Organization for the Research and Treatment of Cancer; HFNC, high-flow nasal cannula; ICU, intensive care unit; IPA, invasive pulmonary aspergillosis; IV, invasive ventilation; N/A, not available; NIV, noninvasive ventilation; PaO_2_/FiO_2_, partial pressure of oxygen in arterial blood/fraction of inspired oxygen; PCT, procalcitonin; SOFA, Sequential Organ Failure Assessment; vv-ECMO, veno-venous extracorporeal membrane oxygenation.

### EORTC-MSG Classification Group

According to our prespecified classification algorithm, 78 patients were classified using EORTC-MSG criteria. Sixty-one (78%) had more than 1 host factor, with a median of 2 host factors per patient (IQR: 2–3) (Table 2, Supplementary Table 1). All patients showed typical morphologic patterns for IPA on computed tomography (CT), with 45 (58%) exhibiting 2 or more CT findings consistent with IPA. At least 1 mycological criterion was positive in 67 (86%) patients, classifying them as probable IPA, while the remaining 11 (14%) were classified as possible IPA due to the absence of an EORTC mycological criterion. All patients classified as possible IPA had serum galactomannan (GM) optical density indices (ODIs) of 0.5 or greater (0.77; IQR: 0.70–0.83) (Table 2, Supplementary Table 2). A total of 22 proven IPA cases (61%) were identified from 36 autopsies, all of them being classified as probable IPA ante mortem. Bronchoalveolar lavage was performed in 61 (78%) patients, with *Aspergillus fumigatus* being the most common species recovered in 39% (30/78) of EORTC-classified patients. Additionally, there was 1 case each of *Aspergillus calidoustus*, *Aspergillus niger*, and *Aspergillus terreus*. Two or more mycological criteria were present in 38 (49%) patients (Table 2). Thus, all patients with EORTC-MSG host factors met the post hoc EORTC-MSG criteria for possible/proven IPA.

**Table 2.** EORTC-MSG Classification Group

EORTC Criteria	No. (%)
Host factor	
Recent history of neutropenia	30 (38%)
Hematologic malignancy	41 (53%)
Receipt of an allogeneic stem cell transplant	19 (24%)
Receipt of a solid-organ transplant	15 (19%)
Prolonged use of corticosteroids	51 (65%)
Treatment with T-cell immunosuppressants	30 (38%)
Treatment with B-cell immunosuppressants	11 (14%)
Inherited severe immunodeficiency	1 (1%)
Acute graft-vs-host disease grade III or IV	7 (9%)
Clinical features	
Dense, well-circumscribed lesion(s) with or without a halo sign	62 (79%)
Air crescent sign	32 (41%)
Cavity	15 (19%)
Wedge-shaped and segmental or lobar consolidation	27 (35%)
Mycological evidence	
*Aspergillus* recovered from sputum, BAL, bronchial brush, or aspirate	33 (42%)
*Aspergillus fumigatus*	30 (39%)
*Aspergillus terreus*	1 (1%)
*Aspergillus calidoustus*	1 (1%)
*Aspergillus niger*	1 (1%)
Galactomannan	
Single serum or plasma: ≥ 1.0	21 (27%)
Single serum or plasma: ≥ 0.5	44 (56%)
BALF: ≥1.0	49 (63%)
Single serum or plasma: ≥0.7 and BALF ≥0.8	2 (3%)
BALF: ≥2 duplicate PCR tests positive	19 (24%)
Classification	
Probable IPA	67 (86%)
Possible IPA	11 (14%)
Diagnostic accuracy	
Percent positive agreement	100%
Percent negative agreement	100%
Overall agreement	100%

This table provides an overview of the classification characteristics for n = 78 patients who were classified according to the EORTC-MSG criteria.

Abbreviations: BAL, bronchoalveolar lavage; BALF, bronchoalveolar lavage fluid; EORTC-MSG, European Organization for the Research and Treatment of Cancer/Mycosis Study Group; IPA, invasive pulmonary aspergillosis; PCR, polymerase chain reaction.

### ICU Classification Group

After confirming the high sensitivity and specificity of the EORTC-MSG criteria in the respective groups, we focused on all non–EORTC-MSG-IPA patients. Invasive pulmonary aspergillosis was diagnosed in 112 ICU patients who did not meet EORTC-MSG host criteria. The most common host factors were COVID-19 in 26 (23%) and influenza in 17 (15%) patients based on FUNDICU criteria. Notably, 50 (45%) of these patients could not be further classified by FUNDICU due to the absence of a proposed host factor. Pathological changes suggesting *Aspergillus* tracheobronchitis were observed in 6 of 112 (5%) patients. A CT scan showing mold-suspicious pathologies was performed in 102 (91%) patients, while 11 (10%) had infiltration observed on chest X-ray only without CT due to hemodynamic or respiratory instability. Mold-suspicious lesions were observed as dense, well-circumscribed areas with or without a halo sign in 19 out of 102 patients (18%), an air crescent sign in 41 patients (40%), cavities in 9 patients (9%), and wedge-shaped, segmental, or lobar consolidation in 33 patients (33%). Bronchoalveolar lavage fluid (BALF) diagnostics were conducted for all patients in this category, each of whom exhibited at least 1 clinical sign or symptom of IPA. The most common *Aspergillus* species recovered from BALF by culture was *A fumigatus* in 71 (63%) patients, followed by 3 cases of *A niger*, and 2 cases each of *Aspergillus flavus* and *A terreus*. Cytology evidence of *Aspergillus* (eg, branching hyphae) was found in only 4 out of 112 cases (4%). Serum GM greater than or equal to 0.5 ODI was positive in 61 (55%) patients, and BALF GM of greater than or equal to 1.0 ODI was positive in 83 (74%) patients. Overall, 59 (53%) patients were classified as probable IPA and/or tracheobronchial aspergillosis according to FUNDICU criteria (Table 3). The remaining 53 patients (47%) could not be classified, with 50 of 112 (45%) lacking defined host factors and 9 of 112 (8%) missing CT scans (6 had both issues). The FUNDICU criteria demonstrated a PPA of 53%, PNA of 100%, and POA of 53%. Most patients with IPA without defined FUNDICU host factors had undergone cardiac surgery with subsequent complications or experienced severe/moderate ARDS due to various causes (Table 4). Using Asp-ICU criteria, 4 (4%) patients were classified as putative IPA, with a PPA of 4%, PNA of 100%, and POA of 4% (Supplementary Table 3). Finally, applying Asp-ICU-BM criteria, 30 (26%) patients were classified as probable IPA (Supplementary Table 4).

**Table 3.** FUNDICU Criteria

FUNDICU Criteria	No. (%)
Host factor	
COVID-19	26 (23%)
Influenza	17 (15%)
Solid tumor	4 (4%)
Uncontrolled HIV infection	1 (1%)
Decompensated cirrhosis	5 (5%)
Moderate/severe COPD	10 (9%)
Compatible signs and symptoms	
Fever persisting after at least 3 d of appropriate antibiotic therapy	17 (15%)
Relapse of fever after a period of at least 48 h of defervescence while still on antibiotics and without other apparent causes	15 (14%)
Pleuritic chest pain	8 (7%)
Pleuritic rubbing of the lungs on examination	3 (2%)
Dyspnea	22 (20%)
Hemoptysis	6 (5%)
Worsening respiratory insufficiency despite appropriate antibiotic therapy and ventilatory support	85 (76%)
Clinical evidence	
Presence of tracheobronchial ulceration and/or nodules and/or pseudo-membrane and/or plaque, and/or eschar on bronchoscopy	6 (5%)
Presence of pulmonary infiltrate(s) by chest CT, or presence of cavitation not attributable to other causes	102 (91%)
Mycological evidence	
Positive *Aspergillus* BALF culture	76 (68%)
*Aspergillus fumigatus*	71 (63%)
*Aspergillus niger*	3 (2%)
*Aspergillus terreus*	1 (1%)
*Aspergillus flavus*	1 (1%)
Galactomannan	
Single serum or plasma: ODI ≥0.5	61 (55%)
BALF: ODI ≥1.0	83 (74%)
Classification	
Probable IPA	53 (47%)
Probable IPA/TBA	3 (2%)
Probable TBA	3 (2%)
Diagnostic accuracy	
Percent positive agreement	53%
Percent negative agreement	100%
Overall agreement	53%

This table provides an overview of the classification characteristics for the 112 patients who were classified according to the FUNDICU criteria.

Abbreviations: BAL, bronchoalveolar lavage; BALF, bronchoalveolar lavage fluid; COPD, chronic obstructive pulmonary disease; COVID-19, coronavirus disease 2019; CT, computed tomography; FUNDICU, Invasive Fungal Diseases in Adult Patients in Intensive Care Unit; HIV, human immunodeficiency virus; IPA, invasive pulmonary aspergillosis; ODI, optical density index; TBA, tracheobroncheal aspergillosis.

**Table 4.** Novel Risk Factors

Risk Factor	No. (%)
Post–complicated cardiac surgery	19 (38%)
Intraoperative massive transfusion (defined as >6 units of packed red blood cells)	19 (100%)
Postoperative pneumothorax	6 (32%)
Postoperative hemothorax	9 (47%)
Postoperative ECMO treatment	10 (53%)
ARDS associated with septic shock (nonpulmonary)	14 (28%)
ARDS (*Streptococcus pneumoniae*)	5 (10%)
OHCA	4 (8%)
Severe pneumonia (severe/moderate ARDS)	5 (10%)
*Orthohantavirus*	2 (40%)
*Legionella pneumophilia*	1 (20%)
*Staphylococcus aureus*	1 (20%)
*Mycobacterium tuberculosis/Landouzy sepsis*	1 (20%)
*Status asthmaticus*	1 (2%)
Acute liver failure	1 (2%)
Asbestosis	1 (2%)

This table provides an overview of host factors that could not be assessed by FUNDICU.

Abbreviations: ARDS, acute respiratory distress syndrome; ECMO, extracorporeal membrane oxygen; FUNDICU, Invasive Fungal Diseases in Adult Patients in Intensive Care Unit; OHCA, out-of-hospital cardiac arrest.

### Improving the Predictive Capacity of ICU Criteria

**Figure 1.**
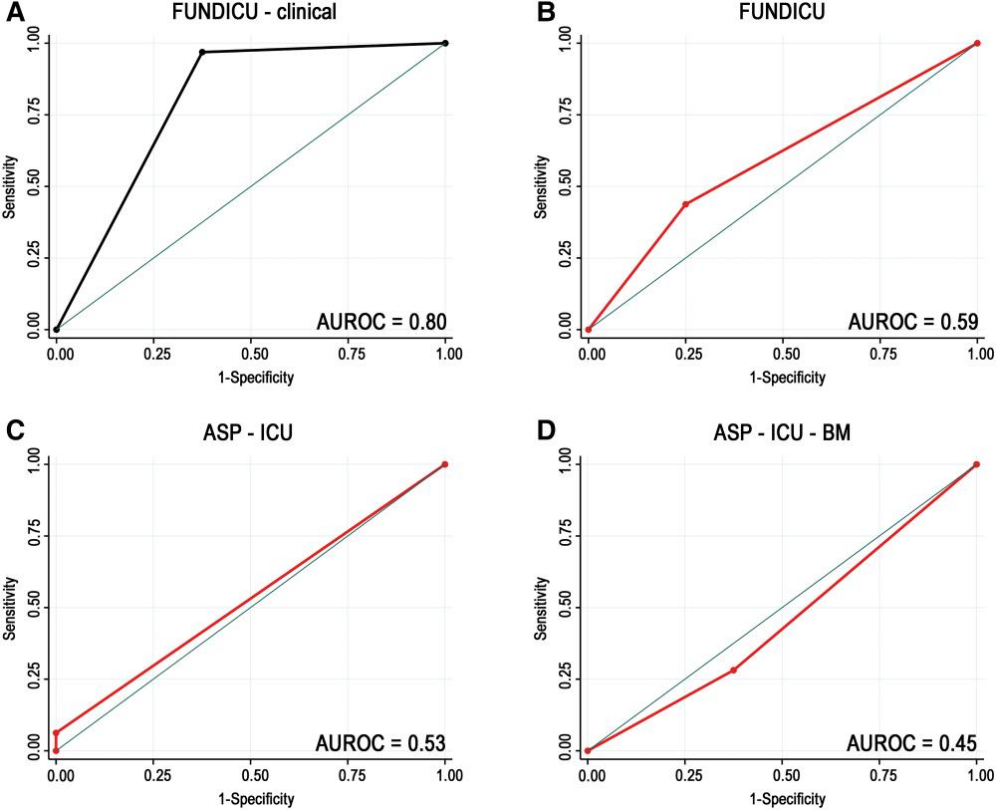
*A–D*, The figure summarizes the diagnostic performance of established algorithms for IPA compared with the novel proposed FUNDICU-clinical algorithm. The performance was evaluated against histologically proven cases, with AUROC used as the measure of accuracy. Abbreviations: ASP-ICU, *Aspergillus* in Intensive Care Units; ASP-ICU-BM, *Aspergillus* in Intensive Care Units with biomarkers; AUROC, area under the receiver operating characteristic curve; FUNDICU, Invasive Fungal Diseases in Adult Patients in Intensive Care Unit; IPA, invasive pulmonary aspergillosis.

We further validated 3 potential diagnostic tools against the reference standard of pathologically proven IPA cases in our ICU cohort (Supplementary Table 5). A total of 40 patients clinically diagnosed with IPA in the non-EORTC ICU cohort had lung biopsy (n = 3) or necropsy (n = 37) and 32 (80%) were confirmed to have IPA by histological examination. Among those histologically confirmed IPA cases, FUNDICU criteria had a sensitivity of 44% and specificity of 75% (PPV = 88%, NPV = 25%). Asp-ICU criteria showed a sensitivity of 6% and specificity of 100% (PPV = 100%, NPV = 22%). Asp-ICU-BM criteria demonstrated a sensitivity of 28% and specificity of 63% (PPV = 75%, NPV = 18%). Since this analysis identified FUNDICU criteria as the best-available diagnostic algorithm for patients with IPA in the ICU we further enhanced FUNDICU by incorporating 2 novel host factors—that is, postoperative complication after cardiac surgery and ARDS. Both post-cardiac surgery and ARDS were identified through an empirical approach, as we studied the population that could not be classified by the FUNDICU criteria and summarized the common denominators, which were post-cardiac surgery and ARDS. This modification of FUNDICU criteria (FUNDICU-clinical) achieved a sensitivity of 97% and specificity of 63% (PPV = 91%, NPV = 83%). FUNDICU-clinical showed significantly better diagnostic performance, with an AUROC of 0.8, compared with FUNDICU (AUROC = 0.59), ASP-ICU (AUROC = 0.53), and ASP-ICU-BM (AUROC = 0.45) (*P* = .022). (Figure 1, Supplementary Figure 6).

### Unclassifiable Group

In the final step of our classification process, we reviewed 12 patients who lacked EORTC-MSG risk factors and were treated outside the ICU. All had potential risk factors for IPA, such as chronic obstructive lung disease, metastatic lung cancer, or interstitial lung disease/fibrosis with long-term low-dose glucocorticoids (<10 mg/d). All patients had CT scans showing mold-suspicious lesions and clinical signs defined in FUNDICU criteria. Each patient had at least 1 mycological finding, leading an ID specialist to clinically classify them as probable IPA, despite lack of any classification system for these patients. Lung biopsies confirmed IPA in 4 out of 4 patients, and necropsies revealed IPA in 2 out of 3 cases. Therefore, 6 out of 7 patients had proven IPA, yielding a true positive rate of 86% (Supplementary Table 6).

## DISCUSSION

In this multicenter observational study, we retrospectively validated the diagnostic performance of various classification tools for diagnosing IPA in 202 patients clinically diagnosed with invasive aspergillosis. We found 100% agreement in patients with traditional host factors who met the EORTC-MSG criteria. However, significant challenges emerged when classifying ICU patients without EORTC-MSG host factors who therefore did not fulfill the EORTC-MSG classification of IPA. Among those patients not classifiable with EORTC-MSG criteria, the latest FUNDICU criteria showed 53% agreement with clinical diagnosis of IPA, leaving nearly half of the patients unclassifiable due to the lack of defined host factors and missing CT scans. The ASP-ICU and ASP-ICU-BM criteria, which typically ignore certain host factors, were significantly less accurate than FUNDICU [11–14]. When evaluated against the gold standard of histopathologically proven IPA, the EORTC-MSG criteria were adequate for classifying patients with traditional risk factors, but ICU systems performed poorly in nonneutropenic ICU patients.

The need for modified classification systems for nonneutropenic ICU patients has been a key focus for the past 25 years, with various research groups tackling this challenge [8, 12, 13, 21]. While ASP-ICU and ASP-ICU-BM are intended as clinical tools for diagnosing IPA in the ICU, FUNDICU was specifically designed for clinical research. Interestingly, FUNDICU outperformed the 2 clinical tools in our cohort, likely due to the stringent mycological criteria used in ASP-ICU and ASP-ICU-BM. Moreover, separating research criteria from clinical decision making is difficult. If we had applied FUNDICU’s research criteria to our IPA patients, 47% would have been excluded, potentially introducing bias by overlooking a large proportion of the at-risk population. While FUNDICU includes several nonclassical risk factors, incorporating additional factors in the future could further enhance its value [22–24]. Given that FUNDICU demonstrates the best diagnostic tool for IPA in ICU patients, we added 2 new categories based on IPA patient profiles—complication after cardiac surgery and moderate/severe ARDS—to FUNDICU’s host factor list. This adjustment significantly improved the diagnostic performance of the updated FUNDICU-clinical algorithm increasing the AUROC from 0.59 to 0.80.

Aspergillosis following major cardiac surgery is a frequently discussed issue, particularly in post-cardiac surgery ICUs, and was added to address these specific challenges [25, 26]. Including ARDS as a category represents a pragmatic approach, as it covers major pulmonary ICU conditions and makes the criteria more adaptable to respiratory viral diseases, including IAPA and CAPA [4, 5].

An interesting aspect regarding gender discrepancy is that a significantly larger proportion of women were classified according to the hematologic-disease–driven EORTC-MSG criteria (47%) compared with the ICU-specific systems (21%). This is likely due to the distribution of host factors among patients with underlying hematological conditions, where there is a trend towards a higher proportion of females. In contrast, the ICU patient group typically exhibits a male-dominant host factor profile (eg, cardiovascular disease or chronic obstructive pulmonary disease).

We identified a group of 12 “unclassifiable” patients who exhibited ICU host factors but were not treated in an ICU, revealing a novel and previously unaddressed at-risk population. This population likely represents an overlooked group of patients with IPA by clinicians, and it is probable that this sample only reflects the “tip of the iceberg.” Future studies should ideally examine this interesting patient group prospectively, with a clear screening approach based on the risk factors we have proposed here.

A key strength of this study is its systematic approach, which included all consecutive patients with IPA from a specific epidemiological area in Austria, minimizing selection bias by using an all-comer population. We used an unbiased method to evaluate all available IPA diagnostic algorithms to identify the most clinically appropriate one. By empirically identifying weaknesses in each algorithm, we aimed to enhance the FUNDICU criteria for greater diagnostic accuracy.

A limitation of the study is its retrospective design, which may introduce information bias based on the clinical assessment of the treating physicians and diagnosis of IPA by ID specialists. Since awareness for IPA may be lower in ICUs compared with hematology wards this could lead to an overrepresentation of patients who fit the EORTC category as well as those with more apparent host factors who rapidly deteriorate after admission. Additionally, using histopathology-controlled cases as the gold standard may introduce bias, as routine biopsies are more likely to be performed in patients with a higher suspicion of or who died from the disease. The proportion of proven IPA cases (33%) aligns with other clinical trials not designed as autopsy studies [8]. Despite this, there was a high rate of confirmed IPA cases (80%) in patients with histological examination of lung biopsies or necropsy clinically judged as IPA cases based on EORTC-MSG or FUNDICU criteria. In patients clinically classified as probable IPA despite any applicable classification system, the confirmation rate was even higher (86%).

In conclusion, the addition of the FUNDICU criteria significantly improved the classification of IPA in ICU patients compared with previously published criteria. By incorporating 2 proposed host factors—postoperative complications after cardiac surgery and ARDS—the diagnostic accuracy can be further enhanced. Additionally, this study provides a basis for future prospective, carefully designed diagnostic studies in this field.

## Supplementary Material

ciae633_Supplementary_Data

## Supplementary Data

Supplementary materials are available at *Clinical Infectious Diseases* online. Consisting of data provided by the authors to benefit the reader, the posted materials are not copyedited and are the sole responsibility of the authors, so questions or comments should be addressed to the corresponding author.

## Notes

***Author contributions*.** S. H. and R. K. designed the study and are responsible for the study implementation. S. H. and R. K. were responsible for data management and quality control of baseline investigation at the study sites. All authors contributed to and approved the final version of the manuscript.

***Potential conflicts of interest.*** R. K. received investigator-initiated research grants from Merck and Pfizer (unrelated to the submitted work) and served as a speaker for Pfizer, Gilead, Astellas, Basilea, Merck, Angelini and Mundipharma. M. H. received research funding from Gilead, Astellas, MSD, IMMY, Pulmocide, Shionogi, Melinta, Mundipharma, Scynexis, F2G, and Pfizer, all unrelated to the submitted work. All other authors report no potential conflicts. Data and analysis code is available on a reasonable request by the first author. All authors have submitted the ICMJE Form for Disclosure of Potential Conflicts of Interest. Conflicts that the editors consider relevant to the content of the manuscript have been disclosed.
