# Supplementary material for: Performance of Diagnostic Algorithms in Patients With Invasive Pulmonary Aspergillosis
Source: Clin Infect Dis. 2024 Dec 20;80(5):1080–7. doi: 10.1093/cid/ciae633 (PMC12135912; doi:10.1093/cid/ciae633)
Supplement: ciae633_Supplementary_Data [file ciae633_supplementary_data.docx]

**Supplementary –** **Performance of diagnostic algorithms in patients with invasive pulmonary aspergillosis**

# Hatzl S. et al.

#
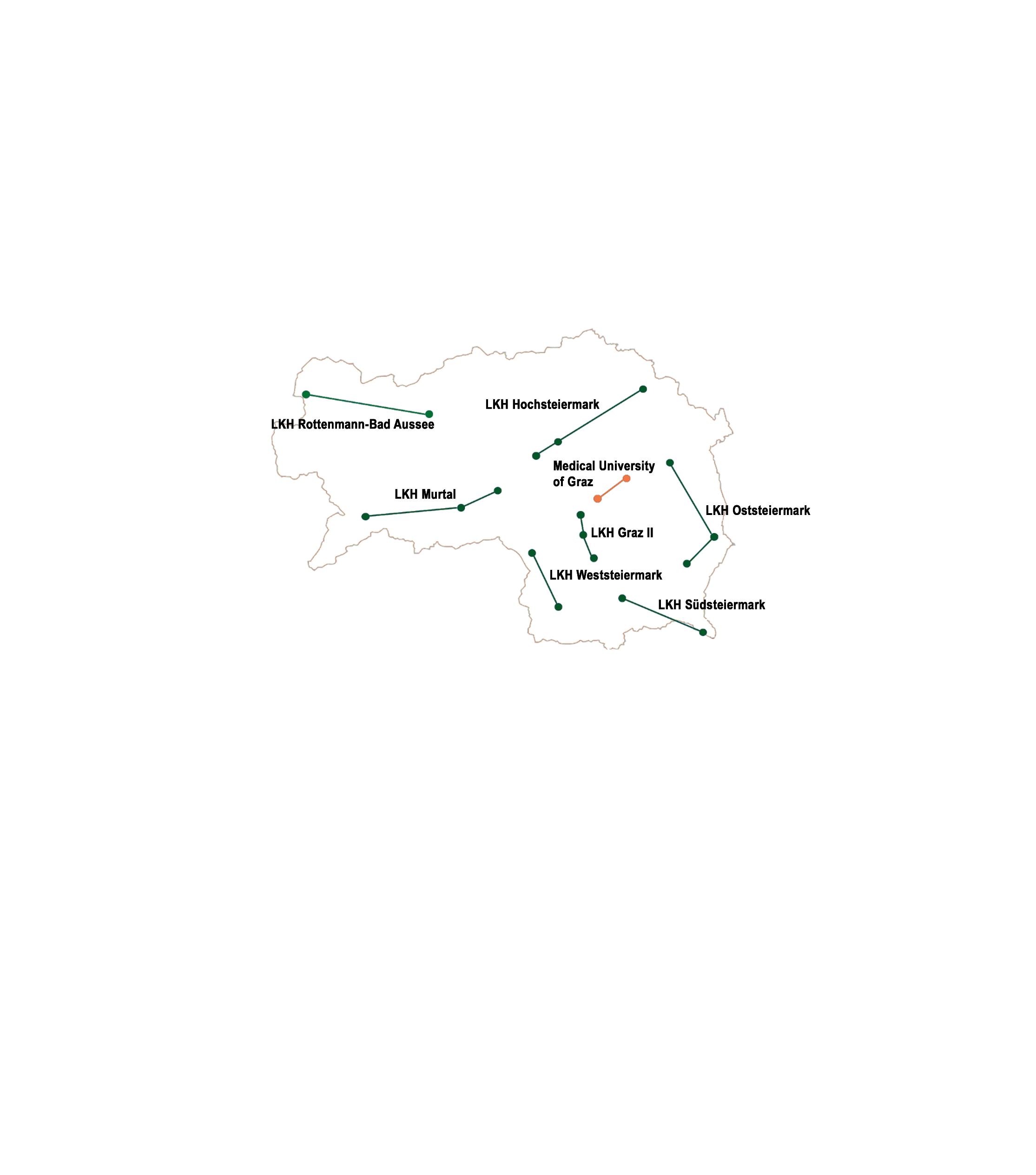


| **Center** | **n** |
| --- | --- |
| LKH-Graz (Internal Medicine) | 131 |
| LKH-Graz (Surgery) | 43 |
| LKH-Graz (Neurology) | 3 |
| LKH-Oststeiermark (Mixed) | 5 |
| LKH-Südsteiermark (Mixed) | 1 |
| LKH-Weststeiermark (Mixed) | 3 |
| LKH-Hochsteiermark (Mixed) | 5 |
| LKH-Graz II (Mixed) | 9 |
| LKH-Rottenmann/ Bad Aussee ((Mixed)) | 2 |

**Supplementary Figure 1: Treatment centers involved in the study**

Each point in the figure refers to a single hospital. The connecting lines show the hospital-networks. LKH – hospital network, n= number of participants enrolled by each center


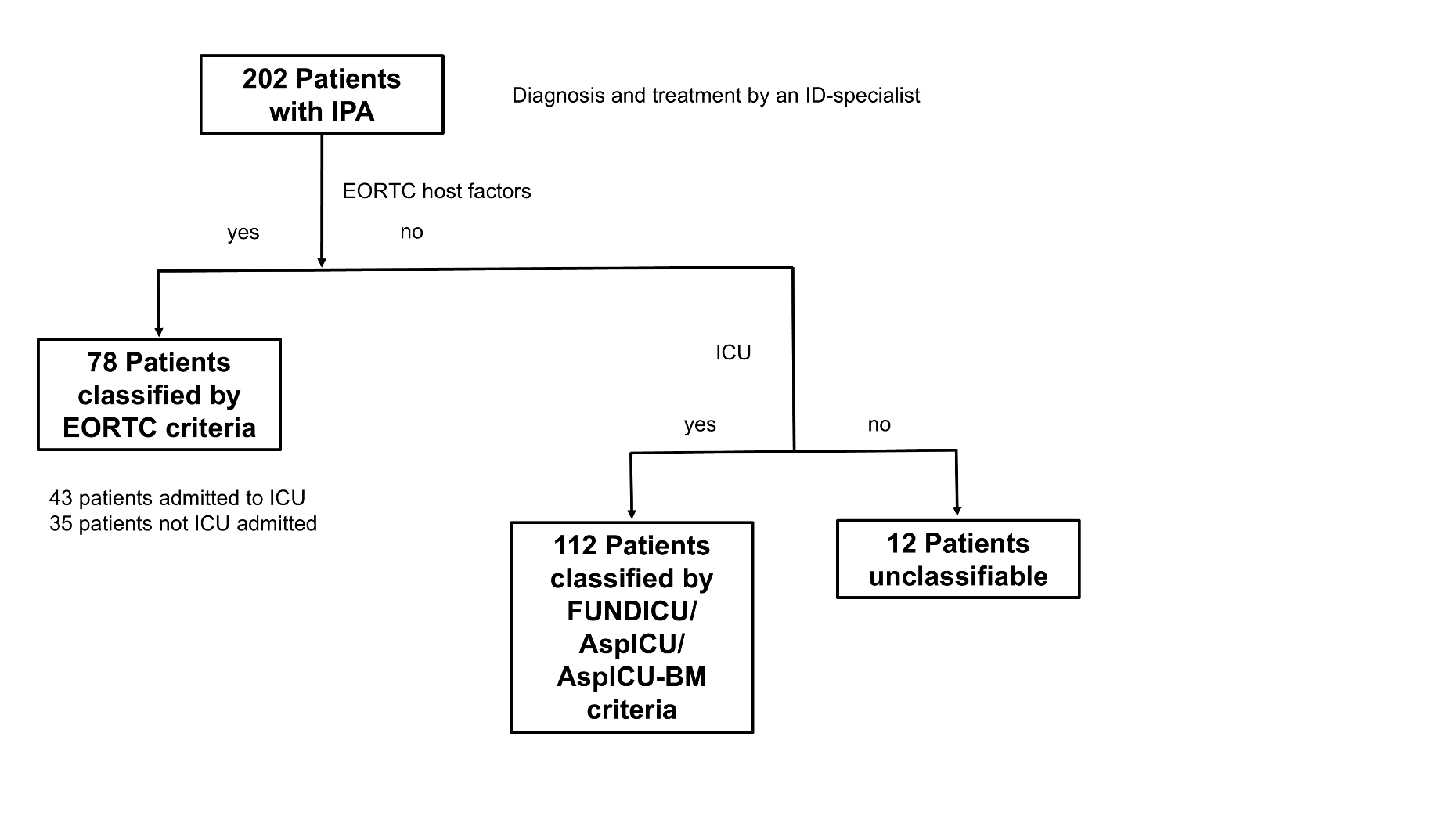


**Supplementary Figure 2: Multistep process of IPA classification**

In the first step we assigned all patients with classical host factors (recent history of neutropenia, hematologic malignancy, allogeneic stem cell transplant, solid organ transplant, and prolonged use of corticosteroids, recognized T-cell immunosuppressant, recognized B-cell immunosuppressant, and severe immunodeficiency, graft - versus - host disease grade III or IV) to classification via EORTC-MSG criteria. (11) These patients were treated in or outside an ICU and did not proceed to the next step. In a second step, we assigned all patients with the diagnosis of IPA within an ICU to classification via FUNDICU, AspICU and AspICU-BM.

IPA – invasive pulmonary aspergillosis; EORTC - European Organization for the Research and Treatment of cancer/Mycosis Study Group; FUNDICU - Invasive Fungal Diseases in Adult Patients in Intensive Care Unit; AspICU - Aspergillosis Intensive Care Unit, AspICU -BM - Aspergillosis Intensive Care Unit biomarkers; ICU – intensive care unit


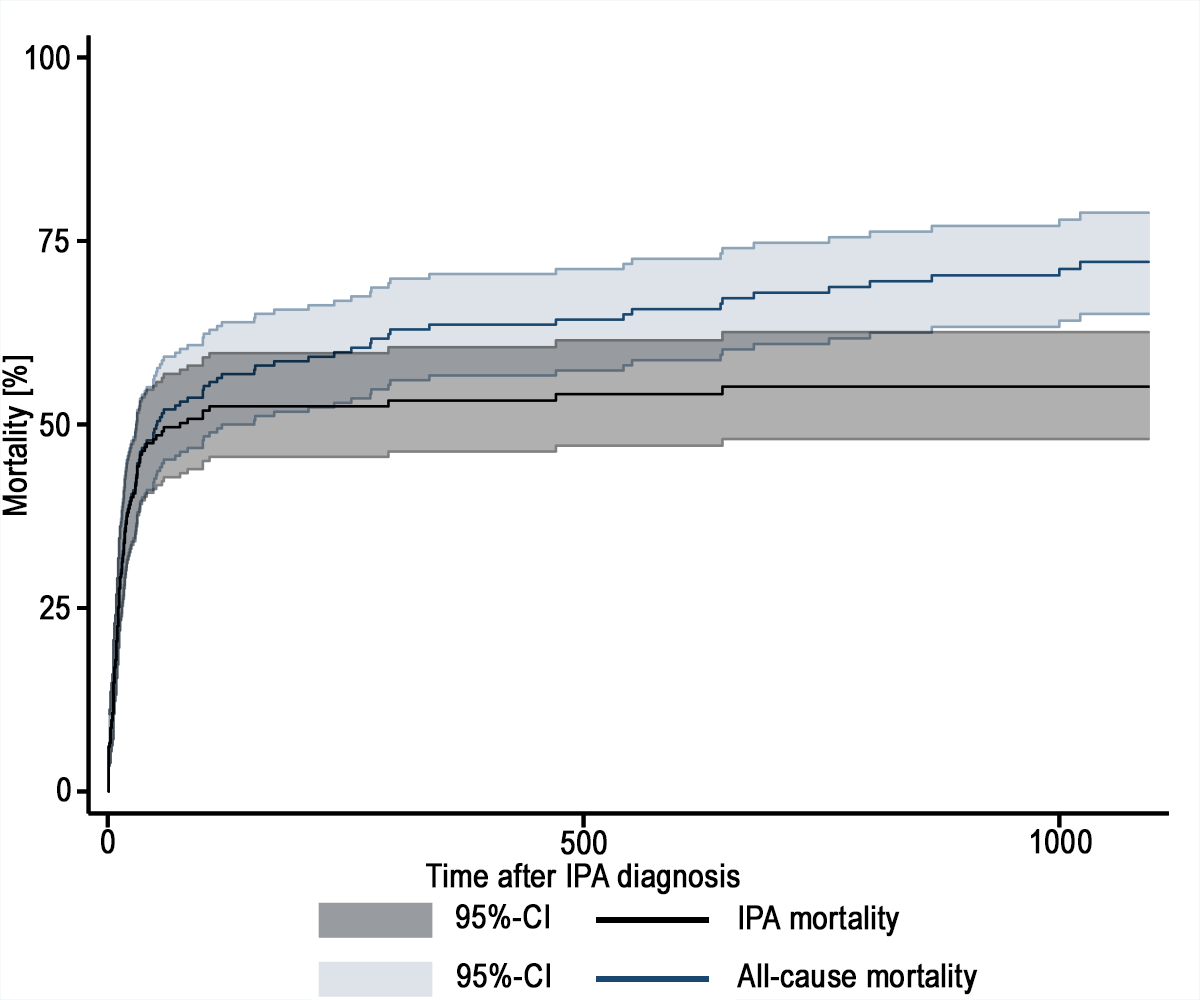


**Supplementary Figure 3: Mortality in the overall cohort**

The figure depicts all-cause mortality (black line) and IPA specific mortality (blue line). X-axis is scaled in days. The shaded areas indicate the 95%-CI. 95%-CI- 95% confidence interval; IPA – invasive pulmonary aspergillosis.


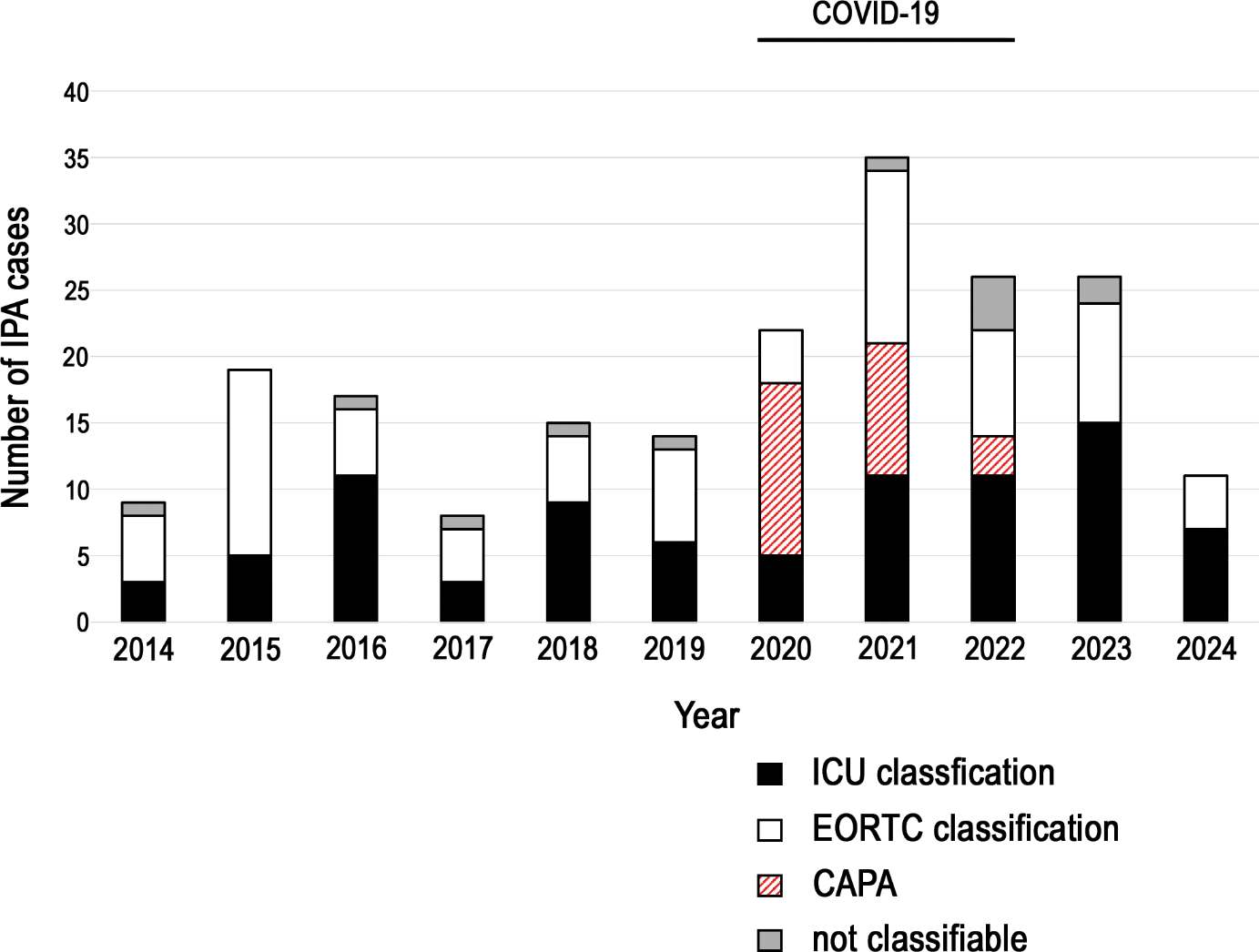


**Supplementary Figure 4: Per year IPA cases according to classification categories**

Black bars depict ICU category, white bars depict the EORTC category, the grey bars depict the unclassifiable category. The red hatched bars show how the COVID-19 pandemic inflates the annual case load by CAPA cases

ICU – intensive care unit; IPA – invasive pulmonary aspergillosis, COVID-19 – coronavirus disease-19; CAPA – COVID-19 associated pulmonary aspergillosis.


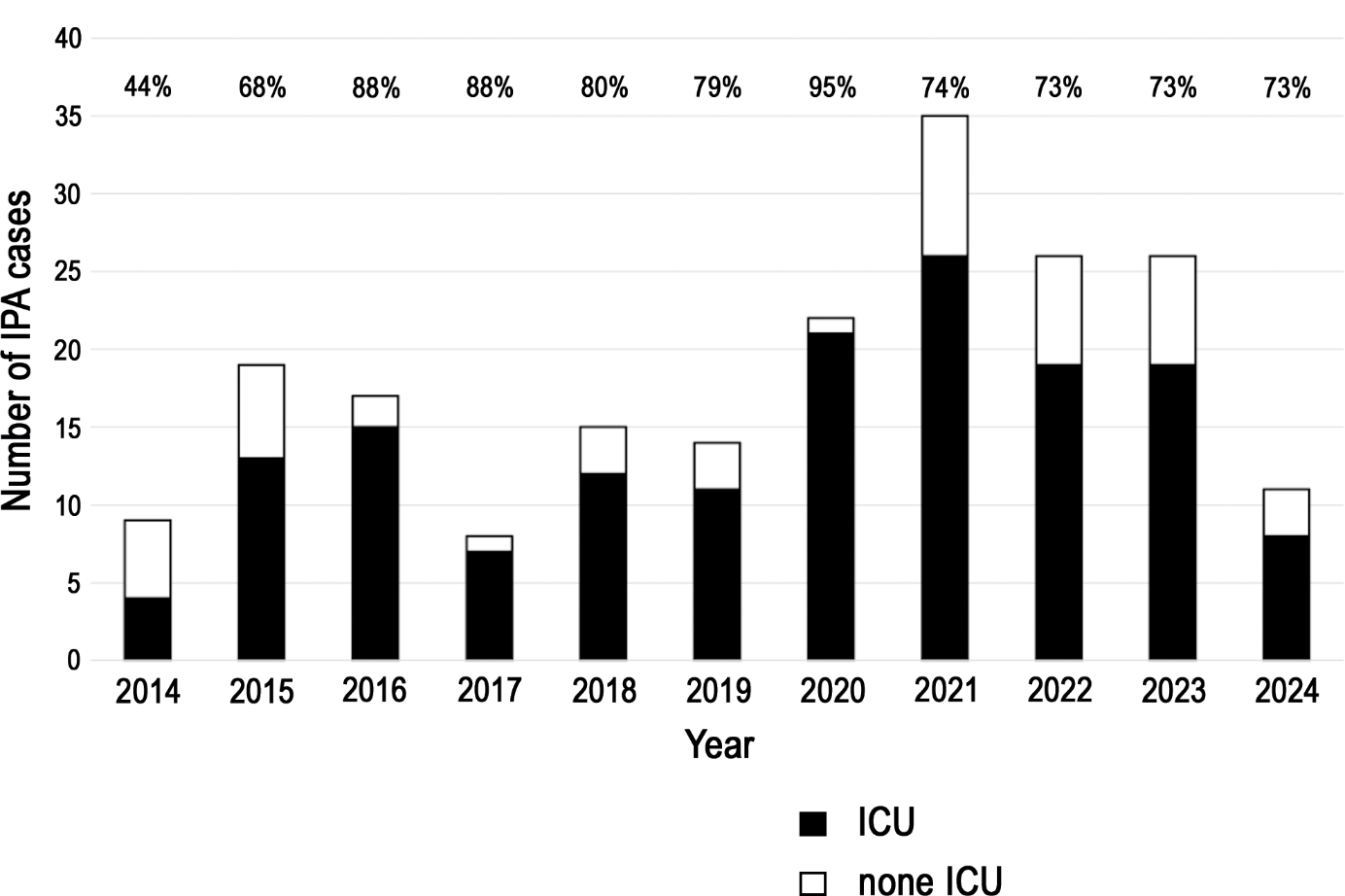


**Supplementary Figure 5: Per year IPA cases according to ICU admission**

Black bars show the number of patients admitted to the ICU irrespective their classification category. White bars show the number of patients treated outside an ICU. The numbers above the bars indicate the percentage of patients admitted to an ICU per year.

ICU – intensive care unit; IPA – invasive pulmonary aspergillosis, COVID-19 – coronavirus disease-19


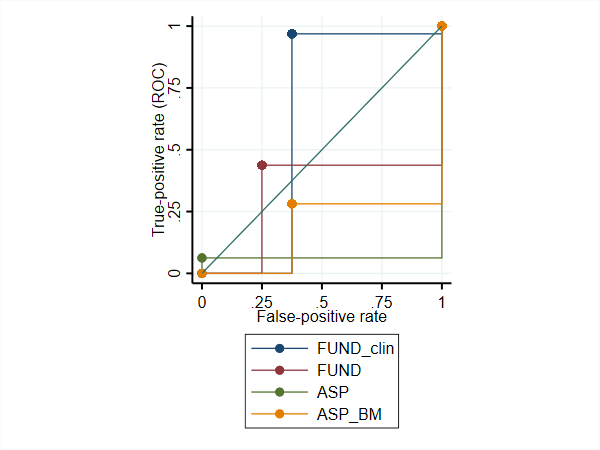


**Supplementary Figure 6: Estimated performance of the ICU scores**

The graph shows the result form the bootstrapped (1000 steps) regression within the receiver operating characteristic of each ICU algorithm. True positive rate is plotted against the false positive rate. FUND_clin – FUNDICU-clinical; FUND -FUNDICU; ASP -ASP -ICU; ASP- BM – ASP-ICU-BM; ROC – receiver operating characteristic

| **Hematologic malignancies** | **n (%)** |
| --- | --- |
| B-cell lymphoid neoplasm | 18 (44%) |
| - Precursor ALL | 7 (39%) |
| - Hodgkin´s lymphoma | 3 (16%) |
| - DLBCL | 3 (16%) |
| - Mantle cell lymphoma | 2 (11%) |
| - Marginal zone lymphoma | 1 (6%) |
| - Multicentric Castleman’s disease | 1 (6%) |
| - Chronic lymphocytic leukemia | 1 (6%) |
| T-cell lymphoid neoplasm | 3 (7%) |
| - Precursor ALL | 1 (33%) |
| - MEITL | 1 (33%) |
| - T-LGL | 1 (33%) |
| Myeloid neoplasm | 17 (42%) |
| - Acute myeloid leukemia | 13 (76%) |
| - Secondary myelofibrosis | 2 (12%) |
| - Myelodysplastic syndrome | 2 (12%) |
| Plasma cell neoplasm | 3 (7%) |
| - Multiple myeloma | 3 (100%) |

**Supplementary Table 1: Hematologic neoplasm in IPA patients (n=41)**

ALL –acute lymphoblastic leukemia; DLBCL – diffuse large B-cell lymphoma; MEITL- monomorphic epitheliotropic intestinal T cell lymphoma; T-LGL – T large granular lymphocytic leukemia.

| **Possible IPA-cases** | **n (%)** |
| --- | --- |
| ***Clinical features*** |  |
| Dense, well-circumscribed lesion(s) with or without a halo sign | 9 (82%) |
| Air crescent sign | 4 (36%) |
| Cavity | 2 (18%) |
| Wedge-shaped and segmental or lobar consolidation | 5 (45%) |
| ***Mycological evidence:*** |  |
| GM: Single serum or plasma: ≥0.5 ODI | 11 (100%) |
| GM: Single serum or plasma: ≥0.7 ODI | 9 (82%) |
| GM: Single serum or plasma: ≥0.7 and BAL fluid ≥0.8 ODI | 2 (3%) |
| GM: BAL fluid 1 PCR tests positive | 6 (54%) |
| Aspergillus lateral flow device (LFD) positive | 5 (45%) |

**Supplementary Table 2: Characteristics of EORTC-MSG possible patients**

This table summarizes the radiologic and mycologic findings of patients meeting the EORTC-MSG possible criteria. Notably, all patients exhibited a galactomannan (GM) value > 0.5 ODI in serum. GM - galactomannan, ODI - optical density index; BAL - bronchoalveolar lavage; PCR -polymerase chain reaction.

| **Asp-ICU - criteria** | **n (%)** |
| --- | --- |
| Aspergillus positive lower respiratory specimen | 76 (68%) |
| - *Aspergillus fumigatus* | 71 (63%) |
| - *Aspergillus niger* | 3 (2%) |
| - *Aspergillus terreus* | 1 (1%) |
| - *Aspergillus flavus* | 1 (1%) |
| Clinical criterion (Fever, pleuritic chest pain, pleuritic rub, dyspnea, hemoptysis, worsening respiratory failure) | 112 (100%) |
| Abnormal medical imaging | 111 (99%) |
| Semiquantitative positive culture (+/++) and absence of bacterial growth | 63 (56%) |
| Cytology evidence of Aspergillus | 4 (4%) |
| ***Classification:*** |  |
| Putative IPA: | 4 (4%) |
| Aspergillus colonization | 13 (12%) |
| ***Diagnostic accuracy:*** |  |
| Percent positive agreement | 4% |
| Percent negative agreement | 100% |
| Overall agreement | 4% |

**Supplementary Table 3: Asp-ICU criteria.**

The table summarizes the characteristic of all patients classified according to the Asp-ICU criteria. IPA – invasive pulmonary aspergillosis

| **Asp-ICU-BM - criteria** | **n (%)** |
| --- | --- |
| Aspergillus positive lower respiratory specimen | 76 (68%) |
| - *Aspergillus fumigatus* | 71 (63%) |
| - *Aspergillus niger* | 3 (2%) |
| - *Aspergillus terreus* | 1 (1%) |
| - *Aspergillus flavus* | 1 (1%) |
| Clinical criterion (Fever, pleuritic chest pain, pleuritic rub, dyspnea, hemoptysis, worsening respiratory failure) | 112 (100%) |
| Abnormal medical imaging | 111 (99%) |
| GM: Single serum or plasma: ODI ≥0.5 | 61 (55%) |
| GM: BAL fluid: ODI ≥1.0 | 83 (74%) |
| Aspergillus PCR (two consecutive PCR´s) | 3 (3%) |
| ***Classification:*** |  |
| Probable IPA: | 30 (26%) |
| Aspergillus colonization | 42 (38%) |
| ***Diagnostic accuracy:*** |  |
| Percent positive agreement | 26% |
| Percent negative agreement | 100% |
| Overall agreement | 26% |

**Supplementary Table 4: Asp-ICU-BM criteria.**

The table summarizes the characteristic of all patients classified according to the Asp-ICU-BM criteria. GM – galactomannan; BAL – broncho alveolar lavage; PCR – polymerase chain reaction; ODI – optical density index; IPA – invasive pulmonary aspergillosis

| **Category** | **Proven IPA n (%)** | **Necropsy/ Biopsy n (%)** |
| --- | --- | --- |
| EORTC-MSG (n=78) | 22 (28%) | 36 (46%) |
| ICU (non-EORTC) (n=112) | 32 (29%) | 40 (36%) |
| unclassifiable | 6 (50%) | 7 (58%) |

**Supplementary Table 5: Overview of proven cases in their respective category**

The table summarizes all proven IPA cases in their respective categories given an overlook EORTC-MSG - European Organization for the Research and Treatment of Cancer/Mycosis Study Group; ICU – intensive care unit

| **Unclassifiable group** | **n (%)** |
| --- | --- |
| ***Host factor*** |  |
| advanced COPD | 4 (34%) |
| Lung carcinoma with metastasis | 3 (25%) |
| Interstitial lung disease/fibrosis (low-dose corticoids) | 3 (25%) |
| Cirrhosis | 1 (8%) |
| Eosinophilic granulomatosis with polyangiitis (low-dose corticoids) | 1 (8%) |
| ***Radiologic signs*** |  |
| Mold suspicious lesions in the CT-scan | 12 (100%) |
| ***Mycological criteria*** |  |
| Aspergillus positive lower respiratory specimen | 9 (75%) |
| - *Aspergillus fumigatus* | 9 (75%) |
| Serum GM ≥ 0.5 ODI | 6 (50%) |
| BAL GM ≥ 1.0 ODI | 6 (50%) |
| BAL Aspergillus PCR positive | 3 (25%) |
| ***Clinical criterion*** |  |
| Clinical criterion (Fever, pleuritic chest pain, pleuritic rub, dyspnea, hemoptysis, worsening respiratory failure) | 12 (100%) |
| ***Classification:*** |  |
| Proven IPA: | 6 (50%) |
| Necropsy | 2 (33%) |
| Lung Biopsy | 4 (66%) |

**Supplementary Table 6: Unclassifiable group**

The table summarizes the characteristic of all patients with unclassifiable host factors.

COPD -chronic obstructive lung disease; ODI – optical density index; BALF – broncho alveolar lavage; IPA – invasive pulmonary aspergillosis; CT – computed tomography
